# Application techniques of a novel hemostat in cardiac operations: HEMOBLAST

**DOI:** 10.1111/jocs.14171

**Published:** 2019-07-26

**Authors:** Brian A. Bruckner, Uy Ngo, Mahesh Ramchandani, Erik Suarez, Samir Awad, Michael Reardon

**Affiliations:** ^1^ Houston Methodist Hospital Methodist DeBakey Heart & Vascular Center Houston Texas; ^2^ Department of Surgery Michael E. DeBakey Veterans Affairs Medical Center Houston Texas

**Keywords:** cardiac, collagen, hemostat, hemostatic powder, thrombin

## Abstract

**Background:**

Postoperative bleeding complications are associated with less favorable outcomes in cardiac surgery and contribute to excessive overall healthcare costs. HEMOBLAST (Biom'up, Lyon, France) (HB) is a novel ready‐to‐use hemostatic powder that consists of porcine collagen, bovine chondroitin sulfate, and human pooled plasma thrombin that may help reduce surgical bleeding.

**Aims:**

The aim of this study was to describe the techniques of application for this new combination powder‐based hemostat, HB, and demonstrate its use employing photographs of application methods during cardiac procedures.

**Materials and Methods:**

The initial 24 procedures in which HB was used at our institution included: left ventricular assist device (LVAD) insertions, lung transplants, heart transplants, aortic valve replacements, coronary artery bypass grafting, and mitral valve repair.

**Results:**

Hemostasis was achieved in all cases and there were no instances of mediastinitis, sternal infections, allergic reactions, or 30‐day mortality.

**Discussion:**

This report describes the best methods of application of HB including use for treatment of mediastinal bleeding in a re‐operative procedure in a patient on antiplatelet agents and sternal bleeding during an LVAD insertion. Proper application can facilitate excellent hemostasis using this powder.

**Conclusion:**

HB is a novel powder‐based multiple component hemostatic agent that promotes focal or large area hemostasis. We have presented the techniques of use that are important to the successful application of HB to facilitate hemostasis.

## 1 INTRODUCTION

Postoperative bleeding complications are associated with less favorable outcomes in cardiac surgery and contribute to excessive overall health care costs.^1^ The need for intraoperative and postoperative blood product administration is also associated with potential risks including adverse reactions, transfusion‐related injuries, or transmission of infectious diseases that are significant factors for morbidity or mortality.^2^ In a study examining open heart procedures requiring re‐exploration for bleeding, it was described that 66% of such cases were from surgical bleeding vs 34% attributed to coagulopathy where no surgical bleeding site could be found.^3^

Topical hemostatic agents have been developed to reduce blood loss within the surgical field and when used as an adjuvant measure in the operating room, may reduce overall bleeding during the postoperative period.^4^ Topical hemostatic agents are usually applied to the bleeding area and pressure is applied to promote clotting and stop active bleeding. A novel combination powder‐based agent HEMOBLAST Bellows (HEMOBLAST; Biom'up, Lyon, France) has now become commercially available and is similar to other powdered hemostats with regard to being readily available and easy to use, but has additional hemostatic properties as it is the only available agent containing chondroitin sulfate and thrombin.^5^ The aim of this study was to describe the techniques of application and use of the new combination powder‐based hemostat (HEMOBLAST) and demonstrate its use employing photographs of application techniques during cardiac procedures.

**Figure 1:**
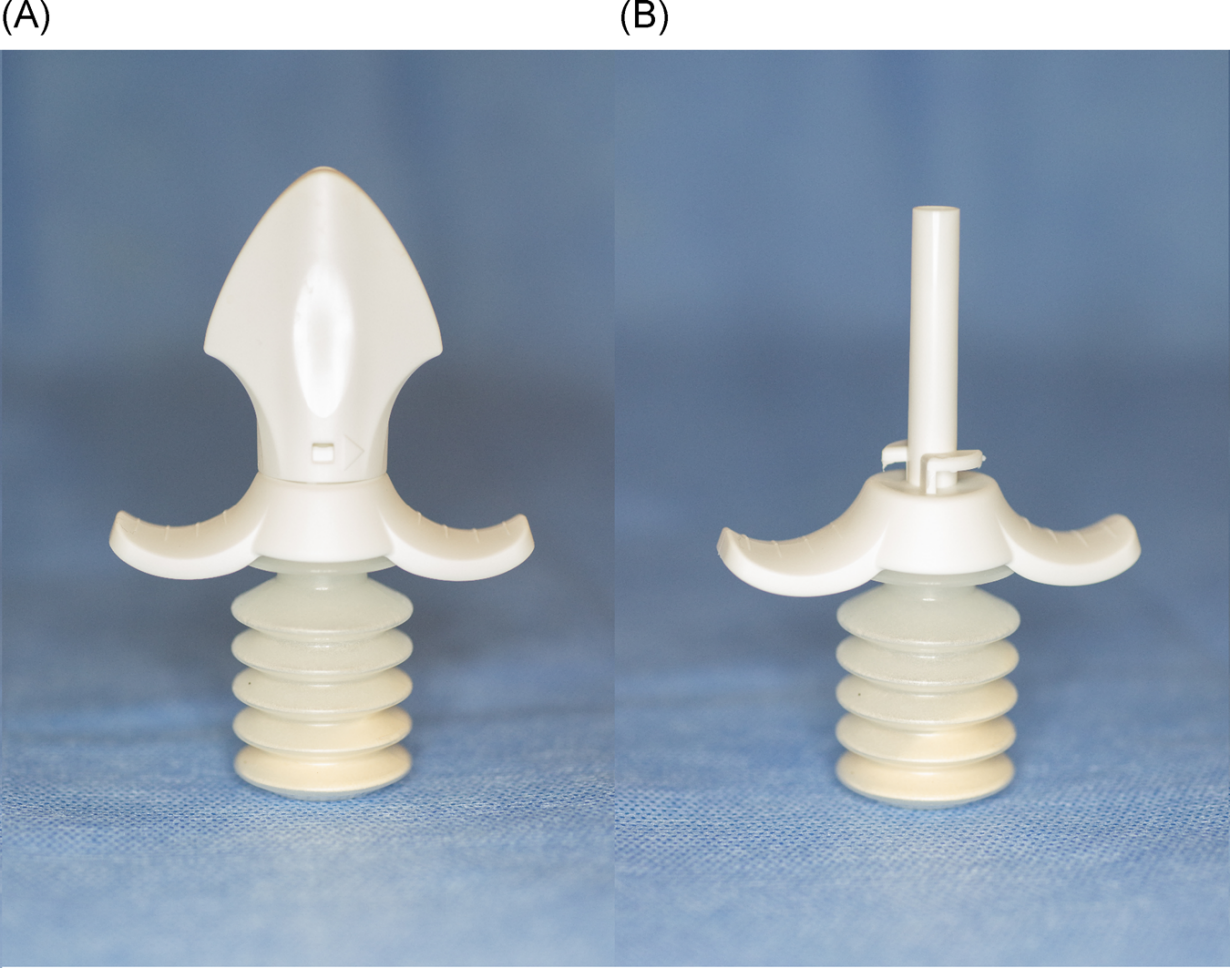
A, HEMOBLAST shown with cap. B, HEMOBLAST shown without cap

HEMOBLAST is a combination agent consisting of porcine‐derived collagen, bovine‐derived chondroitin sulfate, and human‐derived thrombin. The bellows, (Figure 1A and 1B) which contains the hemostat, includes 1.65 g of powder. This device has been evaluated in a recent prospective, randomized, controlled trial, and received the U.S. Food and Drug Administration approval for use in minimal, mild, and moderate bleeding.^6^

## 2 SURGICAL TECHNIQUE

Applying HEMOBLAST first involves quickly blotting excess blood at the target site with a dry pad, sponge, or suction. HEMOBLAST is then rapidly applied to the target site to ensure complete coverage. The powder may be used at a focal bleeding area such as a peripheral vessel, or large diffuse bleeding field such as the mediastinum and heart, including the great vessels. Several applications may be used, especially in revision cardiac cases where dissection of adhesions from previous surgery may lead to diffuse bleeding. After the application of the hemostatic powder, a saline‐soaked laparotomy pad/sponge is then applied and wound appropriate pressure is held for 3 minutes. After pressure is applied, the laparotomy pad is carefully removed to re‐evaluate the surgical field. Gentle irrigation with saline may be used to separate the laparotomy pad from the powder. When applying HEMOBLAST to a vertical surface such as the sternal edge, the laparotomy pad can be placed below the bleeding site to catch falling powder. This powder can then be approximated to the bleeding site when pressure is held with the saline‐soaked pad. If there continues to be bleeding (not controllable by conventional surgical techniques), an additional application of HEMOBLAST can be repeated with the same steps as described above.

## 3 CLINICAL EXPERIENCE

This study was approved by the Institutional Review Board at the Houston Methodist Hospital and patient confidentiality was assured. Patient consent was also obtained for the use of the intraoperative photographs in the manuscript.

For the consented patients undergoing cardiac procedures, the procedures (coronary artery bypass and left ventricular assist device (LVAD) implantation) were performed with the use of full cardiopulmonary bypass which included aortic and atrial cannulation. The patients underwent heparinization and reversal with protamine sulfate at the conclusion of the procedure. HEMOBLAST was applied after protamine administration to the indicated area(s) of the surgical field.

The initial 24 procedures in which HEMOBLAST was used at our institution included: LVAD insertions, lung transplants, heart transplants, aortic valve replacements, coronary artery bypass grafting, and mitral valve repair. With regard to complications occurring in the first 30 days following these cases, no instances of mediastinitis, sternal infections, or allergic reaction were observed. There was no 30‐day mortality in this group of patients.

### 3.1 Bleeding during coronary artery bypass surgery

**Figure 2:**
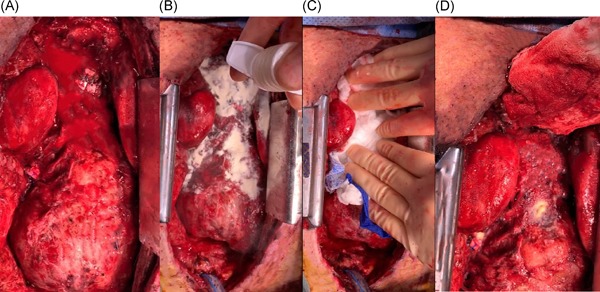
A, Redo sternotomy revealing mediastinal bleeding/oozing. B, HEMOBLAST application to mediastinal bleeding sites. C, Wet lap application to apply pressure. D, Gentle removal of lap pad reveals reduction in bleeding

A 73‐year‐old male underwent a redo coronary artery bypass procedure and had been on preoperative antiplatelet agents including aspirin and clopidogrel. The operative course required extensive dissection of adhesions from the previous bypass procedure and eventually resulted in successful revascularization which included two new saphenous vein grafts to the left‐sided circulation in addition to the already patent left internal artery mammary graft. The case was done utilizing full cardiopulmonary bypass and cardioplegia. After revascularization, the patient was weaned from cardiopulmonary bypass and heparin was reversed with protamine sulfate. The cannulas used for the bypass procedure were removed and the field was inspected for surgical hemostasis, ie, need for polypropylene sutures. After surgical hemostasis had been achieved, diffuse “oozing” in the superior mediastinal area was still noted despite multiple attempts of holding pressure (Figure 2A). HEMOBLAST was applied to this localized area of bleeding where adhesions from a previous thoracotomy were taken down and possibly platelet dysfunction occurred related to preoperative medication and cardiopulmonary bypass. As shown in Figure 2B, once HEMOBLAST was fully applied to the bleeding area, it was followed by the rapid placement of a soaking wet lap pad (Figure 2C). After approximately 3 minutes of gentle manual pressure on the lap pad, HEMOBLAST was slowly removed and gently irrigated. Hemostasis was achieved after removal of the pad (Figure 2D). On closer inspection, it is apparent that there was a transparent adherent layer that had formed over the bleeding site, which resulted in cessation of bleeding/oozing from this area.

### 3.2 Sternal bleeding following LVAD placement

**Figure 3:**
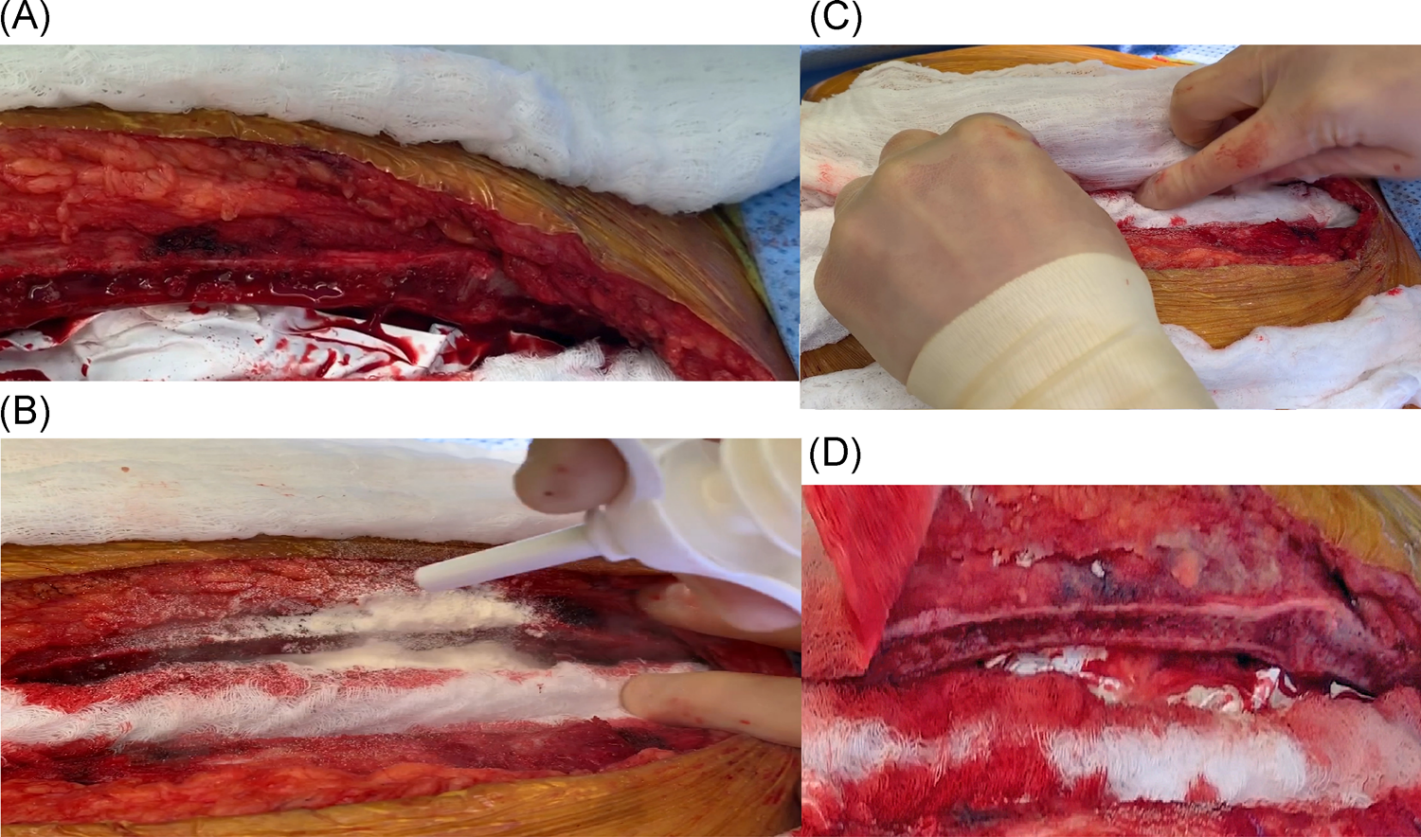
A, Sternal bleeding after LVAD procedure is shown. B, HEMOBLAST applied to bleeding sites on the sternum. C, Wet lap sponge applied for 3 minutes to powder. D, After sponge has been removed significant improvement in hemostasis noted. LVAD, left ventricular assist device

A 57‐year‐old woman with a significant history of end‐stage heart failure underwent LVAD placement. The case was performed utilizing full cardiopulmonary bypass and heparinization. After the device had been placed, the patient was weaned from bypass and the temporary bypass cannulas were removed. Systemic protamine sulfate was given to reverse the heparinization. Before closing the sternal incision, oozing was noted from the sternum and HEMOBLAST was applied for hemostasis. As depicted in Figure 3A, there is an area of “oozing” from the sternal surface. HEMOBLAST was applied in Figure 3B to the sternal surface followed by a soaking wet laparotomy pad (Figure 3C). After 3 minutes of wound appropriate pressure on the wet laparotomy pad, it was removed to reveal improved hemostasis (less oozing of blood from the sternal bone) as demonstrated in Figure 3D.

## 4 COMMENT

Attaining hemostasis during surgery is paramount, whether bleeding is surgical or from an existing coagulopathy, especially during cardiac procedures. Retrospective studies have shown that perioperative red blood cell transfusion is associated with a dose‐dependent increased risk of postoperative cardiac complications, overall morbidity, and in‐hospital mortality.^7^ Additionally, these studies have shown that decreasing the need for transfusions in the perioperative period can lessen the length of intensive care unit stay for patients.^8^ The use of intraoperative hemostatic agents has become increasingly important as they aid with hemostasis and can potentially decrease operating room times, and the need for blood transfusions.^4^, ^9^ The beneficial effects of these agents can contribute to improved patient outcomes and overall health care savings.^4^, ^10^

In this paper, we demonstrate the use of the novel hemostat HEMOBLAST on patients undergoing cardiac surgical procedures. Unlike other powder‐based agents available from our observations in the operative field, the multiple component HEMOBLAST appears to have more adherence properties and actually “sticks” to the field and turns into a thin transparent layer that covers the bleeding site. In addition, the collagen and thrombin would appear to provide strong properties that facilitate platelet aggregation and conversion of fibrinogen to fibrin. Powder‐based hemostatic agents offer the advantage of the ease of use and can cover broad surgical fields or small areas without the need for additional applicators or pressurized gases. These agents are easy to dispense to the surgeon in a matter of seconds and do not require refrigeration or mixing.^6^ From a clinical application prospective, we have used the product in over 60 open cardiothoracic and vascular procedures since late 2018. Improvements in the field of hemostasis have been noted in the majority of these high‐risk patients prone to bleeding (ie, cardiac surgery revisions, LVAD patients, patients on platelet inhibitors, etc.).

In conclusion, HEMOBLAST is a novel powder‐based multiple component hemostatic agent that promotes focal or large area hemostasis. We have presented the techniques of use which are important to the successful application of HEMOBLAST to assist with hemostasis. Additional studies underway will address its effect on surgical blood loss, morbidity, and blood conservation.

## ACKNOWLEDGMENT

The development of this manuscript was supported by Biom'up.

## CONFLICT OF INTERESTS

Dr Bruckner and Dr Awad are consultants of Biom'up. No other authors have relevant conflict of interests to disclose.
